# Dose-volume predictors of post-radiation primary hypothyroidism in head and neck cancer: A systematic review

**DOI:** 10.1016/j.ctro.2022.01.001

**Published:** 2022-01-24

**Authors:** James C.H. Chow, Ka-Man Cheung, Gavin T.C. Cheung, Anthony H.P. Tam, Jeffrey C.F. Lui, Francis K.H. Lee, Kwok-Hung Au, Wai-Tong Ng, Anne W.M. Lee, Harry H.Y. Yiu

**Affiliations:** a Department of Clinical Oncology, Queen Elizabeth Hospital, Hong Kong SAR, China; b Department of Clinical Oncology, Li Ka Shing Faculty of Medicine, The University of Hong Kong, Hong Kong SAR, China; c Comprehensive Oncology Centre, Hong Kong Sanatorium & Hospital, Hong Kong SAR, China; d Department of Clinical Oncology, The University of Hong Kong-Shenzhen Hospital, Shenzhen, China

**Keywords:** 3DRT, dimensional radiotherapy, CI, confidence interval, CT, computer tomography, Dmean, mean dose, Dmin, minimum dose, HNC, head and neck cancer, IMRT, intensity modulated radiotherapy, NPC, nasopharyngeal cancer, NTCP, normal tissue complication probability, OPC, oropharyngeal cancer, OR, odds ratio, ROC, receiver operating curve, TSH, thyroid stimulating hormone, PRIMSA, Preferred Reporting Items for Systematic Reviews and meta-Analyses, QUANTEC, Quantitative Analysis of The Normal Tissue Effects in the Clinic, ULN, upper limit of normal, Vx, thyroid volume that receives x Gy of radiation dose, VSx, thyroid volume that is spared from x Gy of radiation dose, Head and neck neoplasms, Radiotherapy, Hypothyroidism, Survivorship

## Abstract

**Background and Purpose:**

This systematic review aims to identify radiation dose-volume predictors of primary hypothyroidism after radiotherapy in patients with head and neck cancer (HNC).

**Materials and methods:**

We performed a systematic literature search of Medline, EMBASE and Web of Science from database inception to July 1, 2021 for articles that discuss radiation dose-volume predictors of post-radiation primary hypothyroidism in patients with HNC. Data on the incidence, clinical risk factors and radiation dose-volume parameters were extracted. A meta-analysis was performed using the random-effects model to estimate the pooled odds ratio (OR) of thyroid volume as a predictor of the risk of post-radiation hypothyroidism, adjusted for thyroid radiation dosimetry.

**Results:**

Our search identified 29 observational studies involving 4,530 patients. With median follow-up durations ranging from 1.0 to 5.3 years, the average crude incidence of post-radiation primary hypothyroidism was 41.4 % (range, 10 %–57 %). Multiple radiation dose-volume parameters were associated with post-radiation primary hypothyroidism, including the thyroid mean dose (Dmean), minimum dose, V25, V30, V35, V45, V50, V30–60, VS45 and VS60. Thyroid Dmean and V50 were the most frequently proposed dosimetric predictors. The pooled adjusted OR of thyroid volume on the risk of post-radiation primary hypothyroidism was 0.89 (95 % confidence interval, 0.85–0.93; p < 0.001) per 1 cc increment.

**Conclusion:**

Post-radiation primary hypothyroidism is a common late complication after radiotherapy for HNC. Minimizing inadvertent exposure of the thyroid gland to radiation is crucial to prevent this late complication. Radiation dose-volume constraints individualized for thyroid volume should be considered in HNC radiotherapy planning.

## Highlights

• This systematic review included 29 studies (n = 4,530 patients) on dosimetric predictors of primary hypothyroidism in HNC.
• Average crude incidence of primary hypothyroidism after HNC radiotherapy was 41.4%.
• Thyroid Dmean and V50 were the most widely reported dosimetric predictors for hypothyroidism.
• Thyroid volume is a predictor of hypothyroidism (pooled aOR 0.89 per 1 cc increment) independent of radiation dosimetry.
• Thyroid gland constraints individualized for thyroid volume are crucial in HNC radiotherapy.

## Introduction

Radiotherapy is an integral component in the management of localized head and neck cancer (HNC) [1]. Along with advancements in radiotherapy techniques and the surging incidence of human papilloma virus-associated oropharyngeal cancer, an increasing number of patients with HNC are expected to achieve durable disease control [2]. Prevention of late treatment-related toxicity has become a crucial part of survivorship care.

Primary hypothyroidism is a common late endocrine complication of inadvertent radiation injury to the thyroid gland during HNC radiotherapy, and an incidence of 11 %–53 % has been reported across historical series [3]. Compared with other head and neck structures, such as the brainstem and optic chiasm, in which radiation injury is highly debilitating or even lethal, post-radiation hypothyroidism is amendable by lifelong thyroxine replacement. Nevertheless, thyroxine replacement cannot fully mitigate the long-term detriments of hypothyroidism because non-adherence and over- or under-replacement often occur in real-world settings, resulting in up to 30 %–50 % of patients having abnormal thyroid function upon follow-up [4]. Constraining the unintended radiation dose to the thyroid gland remains crucial to minimize the subsequent risk of hypothyroidism in patients with HNC.

Given the relatively inconsequential nature of hypothyroidism, the thyroid gland is often considered as an expendable organ in prospective clinical trials of head and neck radiotherapy [[5], [6]]. International guidelines on HNC radiotherapy planning have designated the thyroid gland as a low-prioritization organ among all normal structures [7]. There is no Quantitative Analysis of Normal Tissue Effects in the Clinic (QUANTEC) report focused on the thyroid gland, but there is increasing interest on the radiation dose-toxicity relationship of this organ in the past decade, as partial sparing of the thyroid gland is feasible, especially with the advent of intensity-modulated radiotherapy (IMRT) [8]. This systematic review aims to provide a comprehensive summary of radiation dose-volume predictors of primary hypothyroidism in patients with HNC and compile data on published clinico-dosimetric models in order to define appropriate constraints for radiotherapy planning.

## Materials and methods

This systematic review was conducted following the Preferred Reporting Items for Systematic Reviews and meta-Analyses (PRISMA) 2020 checklist [9]. We performed a systematic search of Medline, EMBASE and Web of Science from database inception to July 1, 2021 to identify articles that discussed post-radiation hypothyroidism in patients with HNC. The keywords included radiotherapy, hypothyroidism, head and neck cancer and their synonyms or variations. The full search strategy is detailed in Supplementary Table 1. After de-duplication, two authors (JCHC and KMC) independently screened all record titles and abstracts for relevance to the study objectives. Discordant results were resolved by consensus. The exclusion criteria were non-English reports, non-HNC subjects, non-human studies and lack of relevance to radiation-associated hypothyroidism. A full-text review of all potentially eligible articles was performed. Further exclusion criteria included conference abstracts, case reports and review articles; a lack of radiation dose-volume analyses; and secondary hypothyroidism as the study endpoint. Backward searching was performed on the references of included studies and relevant review articles on this topic.

All included studies reported radiation dose-volume analyses of the thyroid gland. The following data were extracted from each eligible study: (1) bibliographic information; (2) patients’ characteristics, including tumor type, radiotherapy technique, use of chemotherapy and follow-up duration; (3) information on post-radiation hypothyroidism, including the latency period, endpoint definitions and follow-up schedules; and (4) the main study results, including the reported incidence of hypothyroidism, clinical risk factors, radiation dose-volume predictors, proposed dosimetric constraints and comparative measures, such as hazard ratios (HRs) and odds ratios (ORs) for the risk of hypothyroidism.

Due to substantial heterogeneity in study methodology and proposed dose-volume constraints, a pooled analysis of the data was not feasible. In this review, various dose-volume predictors of post-radiation hypothyroidism in the literature were summarized qualitatively. A scatter plot was used to present the relationship between the follow-up duration and the crude incidence of post-radiation hypothyroidism. A best-fit line was created using weighted-least squares with log-linear regression. A meta-analysis was performed with the random-effects model to estimate the pooled OR of thyroid volume as a predictor of the risk of post-radiation hypothyroidism. To minimize confounding bias, only studies that reported the OR for thyroid volume (per 1 cc) adjusted for at least one radiation dose-volume parameter were included. A subgroup analysis stratified by the median follow-up duration was performed. The I^2^ statistic and Cochran’s Q test were used to measure heterogeneity. All analyses were performed using RevMan (Version 5.4, The Cochrane Collaboration, 2020).

All of the included studies were systematically assessed for their risk of bias by two independent authors (JCHC and KMC). Any disagreements were resolved by consensus. Eight quality domains were assessed, covering four distinct types of bias in observational studies: (1) selection bias (exclusion of patients with abnormal thyroid function or thyroid diseases before radiotherapy); (2) misclassification bias (exclusion of secondary hypothyroidism, enforcement of regular thyroid function assessment, and adequacy of follow-up duration); (3) reporting bias (systematic evaluation of radiation dose-volume parameters and cut-off values); and (4) confounding bias (whether the proposed radiation dosimetric constraints remained significant after adjustment for clinical factors and thyroid volume).

## Results

### Included studies

**Fig. 1:**
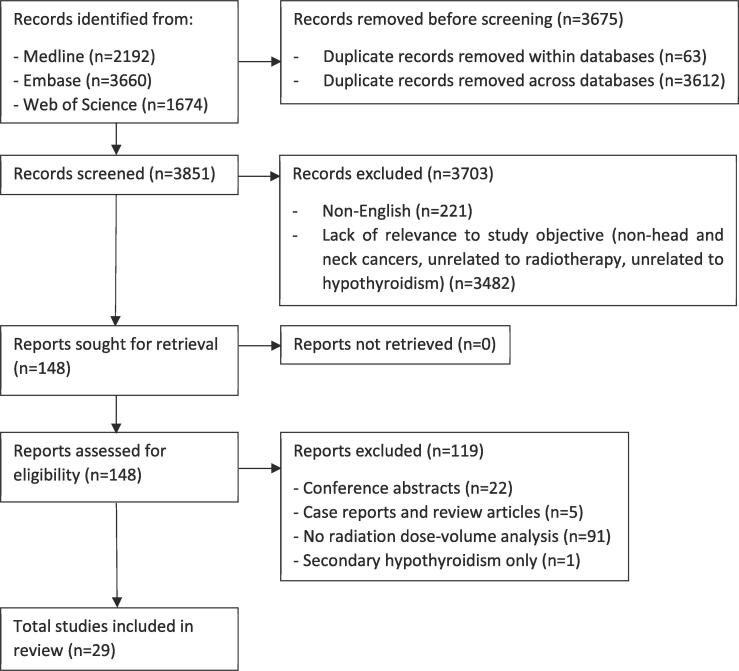
PRISMA flow diagram.

The process of study selection is summarized by the PRISMA flow diagram in Fig. 1. In total, 7,526 articles were identified in the initial literature search. After deduplication, screening and eligibility evaluation, 29 studies involving 4,530 patients were included in this systematic review. The included studies were published from 2010 to 2021 across 13 countries, and all studies were observational in design. Nineteen studies provided comparative data on radiation dose-volume predictors (Table 1) [[8], [10], [11], [12], [13], [14], [15], [16], [17], [18], [19], [20], [21], [22], [23], [24], [25], [26], [27]], while 10 studies reported radiation dosimetric nomograms or normal tissue complication probability (NTCP) models for post-radiation primary hypothyroidism (Table 2) [[28], [29], [30], [31], [32], [33], [34], [35], [36], [37]]. The primary tumors were nasopharyngeal carcinoma (NPC), oropharyngeal carcinoma (OPC), and other sites in 41.4 % (12/29), 17.2 % (5/29), and 41.4 % (12/29) of the studies, respectively. Most patients in the included studies were treated with IMRT.

**Table 1:** Studies on radiation dose-volume parameters of post-radiation hypothyroidism in patients with head and neck cancer.

**Study**	**N for dosimetric analysis**	**Crude incidence of primary hypothyroidism**	**Median FU**	**Tumor types**	**RT technique**	**Chemotherapy**	**Median latency to hypothyroidism**	**Definition for hypothyroidism**	**Proposed dosimetric parameter**	**Proposed cut-off point for dosimetric parameter**	**Relative effect**	**Incidence of hypothyroidism, (low-risk vs high-risk group with reference to the proposed dosimetric cut-off point)**
Diaz 2010	128	61/128 (47.7 %)	2.4 years	HNC	100 % IMRT	100 %	1.1 years	TSH > ULN	None	N/A	N/A	N/A
Kim 2014	114	52/114 (45.6 %)	2.1 years	HNC	44 % IMRT 56 % 3DCRT	57 %	0.7 years	TSH > ULN	V45	<50 %	Continuous, OR 1.02 (CI 1.01–1.03)	1 year, 22.8 % vs 56.1 %
Murthy 2014	43	49/89 (55.1 %)	3.4 years	OP/HP/L	51 % IMRT 49 % 3DCRT	84.%	1.0 years	TSH > ULN	Dmin	<40 Gy	Continuous, HR 1.07 (CI NR, p = 0.012)	Crude, 31.8 % vs 66.7 %
Akgun 2014	100	52/100 (52.0 %)	3.9 years	HNC / Lymphoma	100 % 3DCRT	68 %	NR	TSH > ULN	V30	NR	Continuous, aOR 1.20 (CI NR, p = 0.07)	N/A
Chyan 2014	107	75/123 (61.0 %)	4.6 years	OPC with TV ≥ 8 cc	100 % IMRT	83 %	1.7 years	TSH > ULN and T4 < LLN, or on T4 replacement	VS45	>3cc	NR	3-year, 38 % vs 55 %
Fujiwara 2015	101	39/101 (38.6 %)	2 years	HNC	100 % 3DCRT	NR	1.8 years	TSH > ULN	Dmean	<30 Gy	NR	Crude, 21.9 % vs 56.5 %
Lee 2016	149	54/149 (36.2 %)	3.1 years	NPC	100 % IMRT	77 %	NR	TSH > ULN or T4 < LLN	(i) VS60 (ii) VS45	(i) > 10 cc (ii) > 5 cc	VS60: continuous, aHR 0.70 (CI 0.58–0.86); VS45: continuous, aHR 0.80 (0.73–0.90)	VS60: Crude, 6.0 % vs 30.3 %; VS45: Crude, 6.7 % vs 26.1 %
Ling 2017	102	40/102 (39.2 %)	2.8 years	HNC	95 % IMRT 5 % 3DCRT	26 %	0.7 years	TSH > ULN	(i) V50 (ii) Dmin	(i) < 50 % (ii) < 54.6 Gy	NR	V50: Crude, 12.9 % vs 29.6 %; Dmin: Crude, 15.1 % vs 34.7 %
Sachdev 2017	75	25/75 (33.0 %)	4.2 years	HNC	100 % IMRT	95 %	NR	TSH > ULN and T4 < LLN	V50	<60 %	Dichotomized, OR 6.76 (CI NR, p = 0.002)	NR
Zhai 2017	135	39/135 (28.9 %)	2.8 years	NPC	100 % IMRT	NR	1.3 years	TSH > ULN	(i) Dmean (ii) V45 (iii) V50	(i) < 45 Gy (ii) < 50 % (iii) < 35 %	Dmean: dichotomised, aHR 4.87 (CI 1.72–13.81); V45: dichotomised, aHR 4.59 (CI 1.62–13.02); V50: dichotomised, aHR 5.39 (CI 1.64–17.65)	Dmean: 3-year, 12.5 % vs 58.5 %
Sommat 2017	102	44/102 (43.1 %)	4.1 years	NPC	100 % IMRT	100 %	3.1 years	TSH > ULN	V40	≤85 %	Continuous, aOR, 1.10 (CI 1.02–1.18)	Crude, 21.4 % vs 61.4 %
Xu 2018	52	25/52 (48.0 %)	1.4 years	NPC	100 % IMRT	NR	NR	TSH > ULN, or TSH < ULN with T4 < LLN	(i) Dmean (ii) V50	(i) < 51.6 Gy (ii) < 54.5 %	NR	Dmean: 3-year, 44.6 % vs 67.8 %; V50: 3-year, 29.9 % vs 66.1 %
Lin 2018	56	NR	NR	NPC	100 % IMRT	NR	NR	TSH > ULN	Dmean	<43 Gy	NR	NR
Lertbutsayanukul 2018*	178	96/178 (53.9 %)	3.5 years	NPC	100 % IMRT	100 %	1.8 years	TSH > ULN	VS60	>10 cc	Dichotomized, aHR 0.55 (CI 0.36–0.83)	3-year; 49.2 % vs 66.5 %
El-Shebiney 2018	78	33/78 (42.3 %)	2.6 years	HNC	100 % 3DCRT	81 %	NR	TSH > ULN	V30	<42.1 %	NR	3-year; 29.4 % vs 71.4 %
Huang 2019	345	152/345 (44.1 %)	3.7 years	NPC	100 % IMRT	85 %	NR	TSH > ULN	V25, V35 **and** V45	V25 < 60 %, V35 < 55 % **and** V45 < 45 %	1–2 criteria met vs all 3 criteria met: aHR 2.12 (CI 1.27–3.52); All criteria unmet vs all criteria met. aHR 3.00 (CI 1.78–5.06)	2-year, 13.2 % (all criteria met) vs 24.3 % (1–2 criteria met) vs 36.0 % (all criteria unmet)
Lin 2019	34	36/77 (46.8 %)	3.3 years (euthyroid), 4.7 years (hypothyroid)	HNC	64 % IMRT 36 % 3DCRT	75 %	NR	TSH > ULN	V50	<75 %	Dichotomized, OR 5 (CI 1.03–25)	NR
Zhou 2020	206	104/206 (50.5 %)	1.6 years	NPC	100 % IMRT	94 %	NR	TSH > ULN	V50	24 %	Dichotomized, aOR 8.93 (0.83–89.76)	Crude, 29.0 % (TV < 12.8 cc and V50 ≤ 24 %) vs 39.8 % (TV > 12.8 cc and V50 > 24 %) vs 50.0 % (TV ≤ 12.8 cc and V50 ≤ 24 %) vs 79.0 % (TV ≤ 12.8 cc and V50 > 24 %)
Peng 2020	545	277/545 (50.8 %)	3 years	NPC	100 % IMRT	87 %	2.0 years	TSH > ULN	V30-60	V30-60 ≤ 80 %	Continuous per 10 %, aOR 1.16 (CI 1.05–1.27)	2-year, 11.7 % (TV > 20 cc) vs 19.9 % (TV ≤ 20 cc and V30-60 ≤ 80 %) vs 36.8 % (TV ≤ 20 cc and V30-60 > 80 %)

*External validation study.

Abbreviations: 3DCRT, 3-dimensional conformal radiotherapy; aHR, adjusted hazard ratio; aOR, adjusted odds ratio; cc, cubic centimeter; CI, 95 % confidence interval; Dmin, minimum dose; Dmean, mean dose; FU, follow-up; HNC, head and neck cancer; HR, hazard ratio; HP, hypopharynx; IMRT, intensity-modulated radiotherapy; L, larynx; N, sample size; N/A, not applicable; NPC, nasopharyngeal carcinoma; NR, not reported; OP, oropharynx; OPC, oropharyngeal carcinoma; OR, odds ratio; ROC, receiving operating characteristic; T4, thyroxine; TSH, thyroid stimulating hormone; TV, thyroid volume; ULN, upper limit of normal; Vx (volume of thyroid gland received × Gy); VSx (volume of thyroid spared from × Gy).

**Table 2:** Studies on NTCP models or radiation dosimetric nomograms of post-radiation hypothyroidism in patients with head and neck cancers.

**Study**	**N**	**Hypothyroidism N (%)**	**Median FU**	**Tumor types**	**RT technique**	**Chemotherapy**	**Median latency to hypothyroidism**	**Definition for hypothyroidism**	**Predictive parameter**
**Original NTCP models**									
Bakhshandeh 2012	65	29/65 (44.6 %)	1 year	HNC	100 % 3DCRT	51 %	0.6 years	TSH > ULN	Dmean
Boomsma 2012	105	35/105 (33.3 %)	2.5 years	HNC	33 % IMRT 67 % 3DCRT	14 %	NR	TSH > ULN	TV and Dmean
Ronjom 2013	203	35/203 (17.2 %)	2.1 years	OC/OPC/HP/L	NR	22 %	NR	TSH > ULN	TV and Dmean
Luo 2018	174	39/174 (22.4 %)	2 Years	NPC	82 % IMRT 18 % 3DCRT	89 %	0.8 years	TSH > ULN	Sex, chemotherapy, V50 and pituitary Dmax
**External validation of NTCP models**									
Ronjom 2015	198	19/198 (9.6 %)	1.9 years	OC/OPC/HP/L	NR	39 %	NR	TSH > ULN	External validation of NTCP model by Ronjom 2013
Nowicka 2020	108	31/108 (28.7 %)	2.3 years	OPC	100 % IMRT	25 % induction 60 % concurrent	1.3 years	CTCAE ≥ Grade 2	External validation of NTCP models by Boomsma 2012, Bakhshandeh 2012, and Ronjom 2013
Kamal 2020	360	233/360 (64.7 %)	NR	OPC	100 % IMRT	98 %	1 year	On T4 replacement	External validation of NTCP models by Boomsma 2012
**Nomograms**									
Luo 2017	164	38/164 (23.2 %)	2 years	NPC	80.5 % IMRT 19.5 % 3DCRT	88 %	0.9 years	TSH > ULN	Sex, chemotherapy and V50
Prpic 2019	156	70/156 (44.9 %)	1.9 years	HNC	100 % 3DCRT	55 %	NR	TSH > ULN	TV and Dmin
Zhu 2021	244	138/244 (56.6 %)	5.3 years	NPC	100 % IMRT	82 %	1 year	TSH > ULN	Age, sex, TV and V35 (EQD2 at alpha/beta ratio of 3)

Abbreviations: 3DCRT, 3-dimensional conformal radiotherapy; CTCAE, common terminology criteria for adverse events; Dmax, maximum dose; Dmean, mean dose; EQD2, equivalent dose at 2 Gy per fraction; FU, follow up; HNC, head and neck cancer; IMRT, intensity modulated radiotherapy; N, sample size; N/A, not applicable; NR, not reported; NPC, nasopharyngeal carcinoma; NTCP, normal tissue complication probability; OPC, oropharyngeal carcinoma; T4, thyroxine; TSH, thyroid stimulating hormone; TV, thyroid volume; ULN, upper limit of normal.

In 23 of 29 (79 %) included studies, hypothyroidism was defined as a serum thyroid-stimulating hormone (TSH) level above the upper limit of normal (ULN) in local laboratories [[8], [10], [24], [31], [32], [12], [13], [14], [15], [17], [18], [19], [20], [21], [22], [26], [27], [28], [29], [34], [35], [36], [37]]. The definitions of hypothyroidism in the remaining studies included both a high TSH level and a low serum free thyroxine level [[11], [23]], either a high TSH level or a low free thyroxine level [16], use of thyroxine replacement [[11], [30]] and hypothyroidism classified as grade ≥ 2 according to the Common Terminology Criteria for Adverse Events [33].

The overall risk of bias was low to moderate considering that all of the included studies were observational in design (Supplementary Fig. 1). Of the 29 studies, 56 % and 66 % clearly excluded patients with abnormal pre-radiotherapy thyroid function and a history of thyroid disease, respectively. Most studies clearly excluded patients with secondary hypothyroidism (i.e., low TSH and free thyroxine level). Given the long latency of radiation-associated primary hypothyroidism, 38 % of the studies had a median follow-up duration that exceeded the arbitrary cut-off duration of at least 3 years. Most studies systematically evaluated various radiation dose-volume parameters with regard to hypothyroidism risk. Among the studies that evaluated dose-volume constraints, the proposed cut-off values were derived systematically in 50 %. Half of the included studies evaluated radiation dosimetric parameters with multivariable adjustment for clinical factors and/or thyroid volume.

### Incidence of post-radiation hypothyroidism

**Fig. 2:**
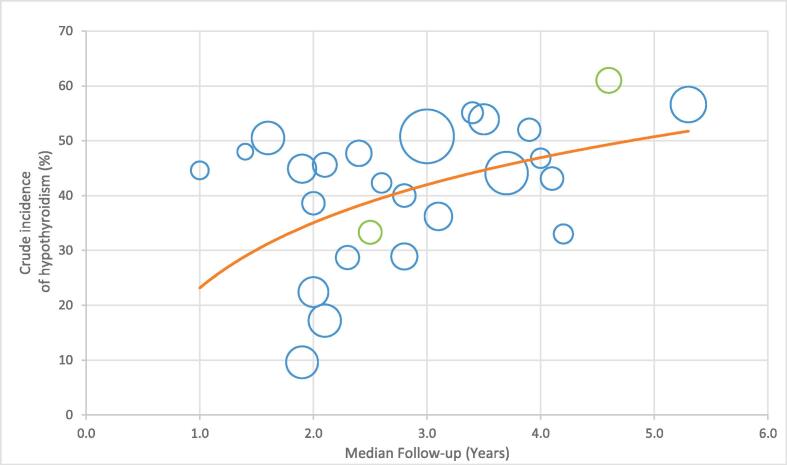
The relationship of crude incidence of post-radiation hypothyroidism and median follow-up duration of individual studies. Each circle represents an independent study (n = 27, data not available in 2 studies). Circle size is proportional to study sample size. Studies that reported incidences of clinical hypothyroidism (high TSH level and low T3/T4 level) were highlighted in green.

The crude incidence of post-radiation hypothyroidism was reported in 27 of 29 studies [[8], [10], [11], [12], [13], [14], [15], [16], [17], [18], [20], [21], [22], [23], [24], [25], [26], [27], [28], [29], [31], [32], [33], [34], [35], [36], [37]]. Patient samples overlapped in two studies; thus, we included the study with the larger sample size only in incidence analyses [[31], [32]]. With a median follow-up duration ranging from 1.0 to 5.3 years, the average crude incidence of post-radiation hypothyroidism was 41.4 % (range, 10 %–57 %). Weighted for study sample sizes, the crude incidence was positively correlated with the median follow-up duration (Fig. 2). The crude incidence of post-radiation hypothyroidism exceeded 30 % in 11 studies in which the median follow-up duration was longer than 3 years [[10], [14], [17], [18], [20], [21], [22], [23], [24]]. Sixteen of 29 studies (55 %) reported the median time to hypothyroidism [[8], [11], [13], [15], [17], [24], [26], [28], [37], [20], [21], [22], [30], [31], [32], [33]]. Hypothyroidism occurred at a median of 1.1 years (range, 0.7–3.1 years) after head and neck radiotherapy.

### Radiation dose-volume predictors

The relationships between thyroid radiation dose-volume parameters and the subsequent risk of hypothyroidism were reported in 19 studies [[8], [10], [11], [12], [13], [14], [15], [16], [17], [18], [19], [20], [21], [22], [23], [24], [25], [26], [27]]. In all studies, thyroid dosimetry was evaluated using computed tomography (CT)-based radiotherapy planning systems. A wide range of thyroid dose-volume parameters was associated with post-radiation hypothyroidism, including V25 (percentage of thyroid volume that received a dose of 25 Gy), V30, V35, V45, V50, V30–60, VS45 (volume of thyroid spared from a dose of 45 Gy), VS60, minimum dose (Dmin) and mean dose (Dmean).

Diaz et al. reported the first CT-based radiation dosimetric study of the thyroid gland in patients with HNC in 2010. Although no significant dosimetric difference was observed between euthyroid and hypothyroid patients, by applying thyroid constraints (V20 < 20 %, V30 < 10 %, V40 < 5 % and Dmax < 50 Gy) during IMRT optimization, lower thyroid doses were achievable than those achieved with unconstrained conventional three-dimensional radiotherapy (3DRT).

Four studies proposed Dmean as a dosimetric predictor of post-radiation hypothyroidism in patients with HNC. Fujiwara et al. evaluated thyroid gland dosimetry in 101 patients with HNC who underwent 3DRT. At a median follow-up duration of 2 years, these researchers observed an incremental incidence of hypothyroidism with thyroid Dmean. The incidence was 21.9 %, 33.3 %, 46.7 % and 55.6 % with Dmean of < 30 Gy, 30–40 Gy, 40–50 Gy and > 50 Gy, respectively [13]. In another study of 135 IMRT-treated patients with NPC, the risk of hypothyroidism was significantly lower in patients with thyroid Dmean < 45 Gy after adjustment for age, sex, thyroid volume and history of thyrotoxicosis [26]. Xu et al. also reported a positive univariable association between thyroid Dmean and the risk of post-radiation hypothyroidism in IMRT-treated NPC patients. The 3-year hypothyroidism risks were 44.6 % and 61.4 %, for thyroid Dmean below and above the threshold of 51.6 Gy, respectively [25]. Similarly, in a small study of 56 patients with NPC, >80 % of patients with hypothyroidism had a thyroid Dmean above 43 Gy [19].

Ten studies have proposed volume-based parameters ranging from V30 to V50 as predictors of post-radiation primary hypothyroidism [[10], [12], [15], [18], [20], [23], [24], [25], [26], [27]]. Of these, two studies recommended minimizing V30 when using 3DRT to treat HNC [[10], [12]]. In one study, the proportion of patients with a thyroid V30 of 100 % was greater in the hypothyroid group than in the euthyroid group (78.3 % vs 59.6 %) [10]. This factor, however, failed to remain significant after adjustment for clinical characteristics. In another smaller study in a similar population, thyroid V30 was correlated with the risk of hypothyroidism, with 3-year incidence rates of 29.4 % and 71.4 % for a V30 < 42.1 % and ≥ 42.1 %, respectively [12].

V40 was predictive of primary hypothyroidism after IMRT in one study [24]. In a dosimetric analysis that included 102 patients with NPC who underwent chemo-radiotherapy, patients with a thyroid V40 of ≤ 85 % had a significantly lower crude incidence of hypothyroidism than patients with a thyroid V40 of > 85 % (21.4 % vs 61.4 %). Upon adjustment for age and *T*-stage, a 10 % increase in the risk of hypothyroidism was observed per 1 % increase in V40. Conversely, two studies proposed to constrain thyroid V45 to < 50 % in HNC radiotherapy [[15], [26]]. Patients with a thyroid V45 of < 50 % had a significantly lower 1-year incidence (22.8 % vs 56.1 %) of hypothyroidism than those who exceeded this threshold [15], and patients with a thyroid V45 of > 50 % were 4.6 times more likely to develop post-radiation hypothyroidism than those who fulfilled this criterion [26].

By far, the most widely proposed dosimetric predictor of primary hypothyroidism in patients with HNC is thyroid V50 [[18], [20], [23], [25], [26], [27]]. In a study by Ling et al., thyroid gland dosimetry was evaluated in 102 patients with HNC [20]. At a median follow-up of 2.8 years, 12.9 % of patients with a thyroid V50 of < 50 % developed hypothyroidism, compared with 29.6 % of patients with a thyroid V50 of > 50 %, but this difference was not statistically significant (p = 0.072). Subsequently, Sachdev et al. conducted a clinico-dosimetric study of 75 patients with HNC who underwent IMRT [23]. By analyzing the receiver operating characteristic (ROC) curve, an optimal V50 cut-off value of 60 % was proposed. At this cut-off value, hypothyroidism was 6.8 times more likely to develop in patients with a thyroid V50 that exceeded this threshold. In contrast, in two studies that adopted a TSH level greater than the ULN as a definition for hypothyroidism, lower V50 constraints of < 35 % and < 54.5 % were proposed [[25], [26]]. The predictive value of thyroid V50 has also been evaluated in relation to thyroid volume. In a large study of 206 IMRT-treated patients with NPC, a thyroid V50 threshold of 24 % was shown to separate the incidence of post-radiation hypothyroidism into 34.2 % and 54.6 [27]. In patients with a thyroid V50 that exceeded 24 %, the crude incidence of hypothyroidism was markedly higher in those with a smaller thyroid volume than in those with a larger thyroid volume (≤12.8 cc [79.0 %] vs > 12.8 cc [39.8 %]).

Two studies proposed thyroid Dmin as a dosimetric predictor of post-radiation hypothyroidism [[20], [21]]. In a small study of 43 patients with oropharyngeal, hypopharyngeal and laryngeal cancers, the crude incidence of hypothyroidism was 31.8 % and 66.7 % among patients with thyroid Dmin values of < 40 Gy and > 40 Gy, respectively [21]. Such a difference, however, was only evident among patients treated with 3DRT. In contrast, in another study of 102 patients with HNC, 95 % of whom underwent IMRT, 34.7 % of patients with a thyroid Dmin of > 54.6 Gy developed hypothyroidism, compared with only 15.1 % of patients with a Dmin of < 54.6 Gy [20].

Apart from conventional radiation dose-volume parameters, three studies confirmed the value of sparing part of the thyroid gland from certain radiation doses [[11], [16], [17]]. This concept was first studied by Chyan et al., who investigated thyroid dosimetry in 107 IMRT-treated patients with OPC whose thyroid volumes were at least 8 cc [11]. In patients in whom at least 3 cc of thyroid gland volume was spared from radiation at 45 Gy (VS45 > 3 cc), the estimated 3-year hypothyroidism risk was 38 %, as compared to 55 % in the VS45 < 3 cc cohort. Lee et al. reproduced similar findings in a study of 149 IMRT-treated patients with NPC [16]. In their clinico-dosimetric analysis, the mean thyroid volume of the included patients was 20.5 cc. Patients with a thyroid VS45 of ≥ 5 cc and a thyroid VS60 of ≥ 10 cc were more likely to be free from post-radiation hypothyroidism. VS45 and VS60 were independently associated with post-radiation hypothyroidism after adjusting for differences in clinical characteristics, chemotherapy usage and variations in thyroid volume. The discriminatory performance of thyroid VS60 was subsequently confirmed in an external validation study [17]. The 3-year radiation hypothyroidism-free rates in patients with a VS60 of ≥ 10 cc and a VS60 of < 10 cc were 33.5 % and 50.8 %, respectively.

Two large studies suggested using combined dose-volume parameters to predict post-radiation hypothyroidism in patients with NPC. In a comprehensive analysis of thyroid dosimetry in 345 patients, Huang et al. proposed three constraints to be used for NPC radiotherapy planning, including a V25 of < 60 %, a V35 of < 55 % and a V45 of < 45 %. When all three constraints were met, the 2-year prevalence of hypothyroidism was low, at 13.2 %, compared with 36.0 % when all three criteria were violated. In a recent study by Peng et al., an unconventional parameter, V30–60 (percentage of thyroid volume receiving > 30 Gy to ≤ 60 Gy), was shown to be a reliable predictor of post-radiation hypothyroidism in patients with a thyroid volume of < 20 cc. The 2-year incidence of post-radiation hypothyroidism was 19.9 % and 36.8 % in patients with a thyroid V30–60 of ≤ 80 % and > 80 %, respectively.

### Association between thyroid volume and post-radiation hypothyroidism

**Fig. 3:**
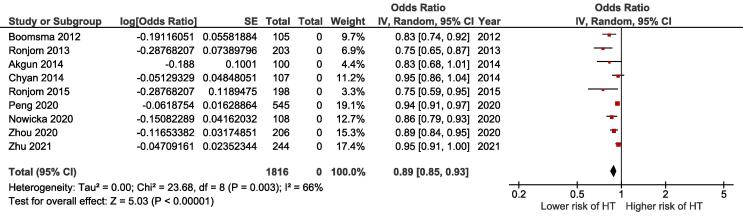
Forest plot showing the association of thyroid volume with post-radiation hypothyroidism. Studies were included only if the reported odds ratios were adjusted for at least one radiation dose-volume parameter in multivariable analyses.

Sixteen studies reported the relative effects of thyroid volume on the risk of post-radiation hypothyroidism adjusted for thyroid dosimetry (Table 3) [[8], [10], [11], [14], [16], [17], [22], [26], [27], [29], [30], [33], [34], [35], [36], [37]]. Of these, 13 studies demonstrated an inverse relationship between thyroid volume and hypothyroidism risk [[8], [14], [16], [22], [26], [27], [29], [30], [33], [34], [35], [36], [37]], whereas 3 studies reported no significant correlation [[10], [11], [17]]. In our meta-analysis of 9 studies (n = 1,816), which reported adjusted ORs for thyroid volume per increment in cubic centimeter, the pooled OR for the risk of hypothyroidism was 0.89 [95 % confidence interval (CI) 0.85–0.93] (Fig. 3) [[10], [11], [22], [27], [29], [33], [35], [36], [37]]. The I^2^ statistic of 66 % indicated presence of significant heterogeneity among the included studies. A subgroup analysis stratified by median follow-up duration was performed (Supplementary Fig. 2). The association of thyroid volume with post-radiation hypothyroidism remained significant in both subgroups (median follow-up of < 3 years: n = 820, pooled OR 0.84, 95 % CI 0.79–0.89; median follow up of ≥ 3 years: n = 996, pooled OR 0.94, 95 % CI 0.92–0.97). The corresponding I^2^ statistics were 37 % (p = 0.17) and 0 % (p = 0.58), respectively.

**Table 3:** Relationship of thyroid volume with post-radiation hypothyroidism in patients with head and neck cancer. Studies were listed only if the reported relative effects were adjusted for at least one radiation dose-volume parameter in multivariable analyses.

**Study**	**Adjusted relative effect measure**	**Relative effect size (95 % CI)**	**p-value**
Diaz 2010	HR	Not reported	<0.05
Boomsma 2012	OR, per cc	0.826 (0.740–0.921)	0.001
Ronjom 2013	OR, per cc	0.75 (0.64–0.85)	<0.001
Akgun 2014	OR, per cc	0.829 (not reported)	0.06
Chyan 2014 #	OR, per cc	0.95 (0.86–1.04)	0.24
Ronjom 2015	OR, per cc	0.75 (0.57–0.90)	<0.001
Lee 2016	HR, per cc	0.889 (0.835–0.951)	0.002
Zhai 2017	HR, ≥16 cc vs < 16 cc	0.290 (0.129–0.653)	<0.003
Lertbutsayanukul 2018	HR, ≥8cc vs < 8 cc	0.80 (0.43–1.49)	0.486
Huang 2019	HR, >16 cc vs ≤ 16 cc	0.517 (0.371 – 0.720)	<0.001
Prpic 2019	OR, per log(cc)	0.312 (0.112–0.868)	0.026
Zhou 2020	OR, per cc	0.89 (0.83–0.94)	<0.001
Peng 2020	OR, per cc	0.94 (0.91–0.97)	0.001
Nowicka 2020	OR, per cc	0.86 (0.79–0.93)	<0.001
Kamal 2020*	Not reported	Not reported	<0.001
Zhu 2021	OR, per cc	0.954 (0.911–0.999)	0.046

#Analysis excluded patients with thyroid volume < 8 cc.

*Bayesian Information Criteria-minimizing stepwise forward model was used, false discovery rate logworth 12.326.

*Abbreviations: cc, cubic centimeter; CI; confidence interval; HR, adjusted hazard ratio; OR, adjusted odds ratio;* vs *versus.*

### Association between clinical factors and post-radiation hypothyroidism

Fifteen studies reported associations between clinical factors and post-radiation hypothyroidism with multivariable adjustment for thyroid dosimetry (Supplementary Table 2) [[8], [11], [14], [17], [23], [24], [26], [27], [29], [31], [32], [33], [35], [36], [37]]. Seven studies observed no effect of age on the risk of hypothyroidism [[11], [14], [23], [29], [33], [35], [36]], whereas four studies suggested that hypothyroidism susceptibility is higher in young patients [[8], [24], [26], [37]]. Sex, surgical history, tumor stage and chemotherapy usage were not identified as independent predictors in most reports. Of the four studies that evaluated the pre-radiotherapy TSH level, two studies identified positive associations between a high baseline TSH level and subsequent hypothyroidism risk [[17], [36]]. However, patients with abnormal thyroid function before treatment were not excluded in one study [36], and no adjustment was made for pre-treatment thyroid volume in the other [17].

### NTCP models and prediction nomograms

NTCP models of post-radiation hypothyroidism in patients with HNC were described in four studies (Supplementary Table 3) [[28], [29], [32], [35]]. In a small prospective report of 65 patients who underwent 3DRT for HNC, Bakhshandeh et al. proposed a Lyman equivalent uniform dose mean dose-based model of post-radiation hypothyroidism, which estimated D50 at approximately 44 Gy [28]. Boomsma et al. developed a bivariable NTCP model that included thyroid Dmean and thyroid volume using 2-year follow-up data from a prospective cohort of 105 patients with HNC [29]. At a thyroid Dmean of 45 Gy, the NTCP increased by 5 % for each 1 cc reduction in thyroid volume. Similarly, Ronjom et al. developed a mixture model with latent time correction to estimate the NTCP for post-radiation hypothyroidism using the dosimetric data of 203 patients with HNC [35]. The risk of hypothyroidism was dependent on both thyroid Dmean and thyroid volume; the Dmean thresholds for a 25 % risk of hypothyroidism at thyroid volumes of 10 cc, 15 cc, 20 cc and 25 cc were 26 Gy, 38 Gy, 48 Gy and 61 Gy, respectively. These three models have been externally validated in independent cohorts and demonstrated satisfactory performance in terms of discrimination and calibration, with the exception of the model by Bakhshandeh et al., which demonstrated an accuracy of < 50 % [[30], [33], [36]]. Subsequently, a four-variable NTCP model for post-radiation primary hypothyroidism, which included sex, chemotherapy usage, thyroid V50 and maximum dose to the pituitary gland, was developed for patients with NPC [32]. This model demonstrated a good internal fit, with an area under the ROC curve of 0.793. No external validation study for this model has been identified.

We identified three clinico-dosimetric nomograms for post-radiation hypothyroidism, all of which have demonstrated good internal discrimination with concordance indices of > 0.7 (Supplementary Table 4) [[31], [34], [37]]. In a study of 156 patients with HNC treated with 3DRT, a hypothyroidism risk score based on thyroid volume and thyroid Dmin has been proposed, which demonstrated a sensitivity and specificity of 75.7 % and 62.6 %, respectively [34]. Two other nomograms have been developed specifically for patients with NPC. Luo et al. built a nomogram for post-radiation hypothyroidism that included sex, thyroid V50 and chemotherapy exposure [31]. Satisfactory prediction of thyroid status was consistently observed at 18, 24 and up to 30 months after radiotherapy. Recently, a four-variable nomogram, including age, sex, thyroid volume and V35, was constructed using data from 244 patients with NPC over a median follow-up duration of 5.3 years. This nomogram has demonstrated good internal predictive ability and calibration [37]. However, no external validation studies have been identified for these published nomograms.

## Discussion

This systematic review summarized 29 contemporary studies that reported the association between thyroid radiation dosimetry and the risk of post-radiation hypothyroidism in patients with HNC. We identified two systematic reviews on this topic, both of which were published in 2011. The studies included in these reviews were limited by the use of conventional radiotherapy techniques, in which thyroid dosimetry was approximated by prescription doses, surface doses, or reconstructed treatment plans [[3], [38]]. As CT-based IMRT planning has become the standard for HNC, our review provides a comprehensive update on the dose–effect relationship of the thyroid gland and summarizes potential dosimetric constraints for this organ in modern head and neck radiotherapy.

Radiation-associated hypothyroidism is a common late complication in patients with HNC that manifests over a wide range of latency periods. In this review, we estimated a high average crude incidence of 41.4 % at median follow-up durations of 1.0–5.3 years. A clear uptrend in the reported incidence was observed in studies with long follow-up periods with no clear indication of a plateau, highlighting the importance of regular long-term surveillance of thyroid function in survivors of HNC who undergo neck irradiation. This latency of post-radiation hypothyroidism also raises concerns about bias in observational studies. Given that 62 % of the included studies had a median follow-up duration of < 3 years, a considerable proportion of patients might have been prematurely classified as having euthyroidism, resulting in biased estimations of dosimetric effects, especially when hypothyroidism was analyzed as a binary outcome with no adjustment for time as a factor.

In contrast to a previous review, which concluded no correlation between thyroid dosimetry and hypothyroidism risk [3], almost all subsequent studies in the era of CT-based radiotherapy planning have reported clear dose–effect relationships in the thyroid gland. Constraints using Dmean or volume-based parameters at intermediate doses (e.g. V30–V50) have been commonly proposed. Nevertheless, dose cut-off values for these parameters vary significantly across studies. For instance, in the six studies that confirmed the predictive value of thyroid V50, the cut-off values ranged from 24 % to 75 %. Of note, regardless of the proposed dose-volume parameters and cut-off values, the incidence of hypothyroidism in low-risk patients was invariably >10 % in most studies. Therefore, instead of viewing these thresholds as risk-free constraints, thyroid doses (e.g. V50 or Dmean) should be kept as low as is achievable during HNC radiotherapy planning to minimize the subsequent risk of post-radiation hypothyroidism.

Our study confirmed a significant inverse relationship between thyroid volume and the risk of post-radiation hypothyroidism, independent of thyroid dosimetry. For every 1 cc increase in thyroid volume, the risk of hypothyroidism decreased by 11 %. As thyroid volume is a surrogate for the functional reserve of thyroxine production, it is understandable that patients with a smaller thyroid volume are more vulnerable to radiation-associated primary hypothyroidism. Our finding indicates that a single radiation dose-volume constraint for the thyroid gland is not universally applicable to all patients. Instead, individualized constraints tailored for different thyroid volumes should be developed, with a more stringent threshold for patients with a small thyroid volume. Alternatively, unconventional dose-volume parameters that are less sensitive to differences in thyroid volume, such as VS60, should be considered for radiotherapy planning. Instead of constraining the thyroid volume that receives a certain radiation dose (Vx), sparing a finite volume of thyroid tissue from “thyrocidal” radiation doses (e.g. VS60 > 10 cc) is conceptually more relevant to the maintenance of long-term secretory function.

Historically, certain factors, such as young age, female sex and history of neck surgery or chemotherapy, have been shown to confer a higher risk of post-radiation hypothyroidism in patients with HNC [[38], [39]]. However, many of these reported associations were unadjusted for differences in thyroid dosimetry and thyroid volume. In this review, we deliberately focused on studies that evaluated the effects of clinical factors with adjustment for thyroid radiation dosimetry. No clear independent associations were observed for most factors. Young age appeared to be a possible risk modifier, but a quantitative pooled analysis to determine the effect of age was not feasible due to missing data and heterogeneity in the outcome measures of the included studies. Overall, our observation highlighted radiation injury as the dominant etiology of this complication. Although it remains uncertain whether clinical factors can independently modify the risk of post-radiation hypothyroidism, it is crucial for future studies to include thyroid volume as a covariate when evaluating clinical factors to account for inherent differences in thyroid reserve.

This review has several limitations that should be noted. First, the observational studies included in this review were heterogeneous in terms of patient composition, thyroid function surveillance intensity, robustness of confounder adjustment and choice of effect size measurement. These variations affected the accuracy of hypothyroidism risk estimation and resulted in a wide range of dose-volume constraints; thus, a quantitative summary of existing data was not feasible. Obtaining individual patient data from the published studies would be helpful to generate a representative NTCP model. Second, all of the proposed constraints, nomograms and NTCP models were derived at single institutions; thus, external validation in independent cohorts is lacking. Third, most of the included dosimetric studies defined hypothyroidism using TSH level alone. Although a substantial proportion of patients with subclinical hypothyroidism would eventually develop overt hypothyroidism, the incidence and radiation dose-volume predictors for overt or symptomatic post-radiation primary hypothyroidism remains unclear. Finally, a significant number of the included studies had inadequate follow-up durations to fully capture the risk of post-radiation hypothyroidism. Given the long latency of this complication, significant misclassification bias may be present, rendering the risk predictions only relevant for short-term thyroid function outcomes.

In conclusion, the incidence of post-radiation primary hypothyroidism is high after high-dose radiotherapy for HNC. Our review has confirmed a clear radiation dose-toxicity relationship of the thyroid gland and post-radiation primary hypothyroidism. The risk of hypothyroidism decreases as a function of thyroid volume, which is independent of thyroid dosimetry. Although multiple dose-volume parameters were predictive of post-radiation primary hypothyroidism, thyroid V50 and thyroid Dmean were most extensively studied. Averaging the proposed cut-offs, constraints such as V50 < 50 % and Dmean < 40 Gy could be considered for clinical application, but more stringent cut-offs arerequired for patients with small thyroid glands. Constraints that focus on sparing thyroid reserve, such as VS60 > 10 cc, are also relevant. External validation studies using long-term thyroid function data are needed to confirm the utility of individual constraints and prediction models.

## Patient Consent Statement

This is a systematic review which involved no individual patient data.

## Funding

None.

## Declaration of Competing Interest

The authors declare that they have no known competing financial interests or personal relationships that could have appeared to influence the work reported in this paper.
