# Supplementary material for: Dose-volume predictors of post-radiation primary hypothyroidism in head and neck cancer: A systematic review
Source: Clin Transl Radiat Oncol. 2022 Jan 24;33:83–92. doi: 10.1016/j.ctro.2022.01.001 (PMC8807951; doi:10.1016/j.ctro.2022.01.001)
Supplement: Supplementary data 1 [file mmc1.pdf]

Supplementary Figure 1

Risk of bias assessment of included studies. Green denotes low risk of bias, red denotes high risk of bias, and grey denotes non-applicable domain.

	Selection bias		Misclassification bias			Reporting bias		Confounding bias	
	Exclusion of abnormal pre-radiotherapy TFT	Exclusion of prior thyroid disease	Exclusion of secondary hypothyroidism	Regular TFT monitoring after radiotherapy	Adequate follow-up duration (median >3 years)	Systematic evaluation of DVH parameter(s)	Systematic evaluation of cut-off value(s)	Multivariable adjustment for clinical factor(s)	Multivariable adjustment for thyroid volume
**Studies on radiation dosimetric constraints (n=19)**									
Diaz 2010	Red	Green	Green	Green	Red	Green	Red	Green	Green
Kim 2014	Green	Green	Green	Green	Red	Green	Green	Red	Red
Murthy 2014	Green	Red	Green	Red	Green	Green	Red	Red	Red
Akgun 2014	Green	Red	Green	Green	Green	Green	Red	Red	Green
Chyan 2014	Red	Green	Red	Green	Green	Green	Red	Green	Green
Fujiwara 2015	Green	Red	Green	Green	Red	Green	Red	Red	Red
Lee 2016	Red	Green	Red	Green	Green	Green	Red	Green	Red
Ling 2017	Red	Green	Green	Red	Red	Red	Red	Red	Red
Sachdev 2017	Red	Red	Green	Red	Green	Green	Green	Green	Red
Zhai 2017	Green	Green	Green	Green	Red	Green	Red	Green	Green
Sommat 2017	Red	Red	Green	Green	Green	Green	Green	Green	Red
Xu 2018	Red	Green	Red	Green	Red	Green	Green	Red	Red
Lin 2018	Red	Red	Green	Green	Red	Red	Red	Red	Red
Lertbutsayanukul 2018*	Green	Red	Green	Red	Green	Grey	Grey	Green	Green
El-Shebiney 2018	Green	Green	Green	Green	Red	Green	Green	Red	Red
Huang 2019	Green	Green	Green	Green	Green	Green	Green	Green	Green
Lin 2019	Green	Red	Green	Red	Green	Green	Green	Green	Green
Zhou 2020	Red	Green	Green	Red	Red	Green	Green	Green	Green
Peng 2020	Red	Red	Green	Red	Green	Green	Green	Red	Green

	Selection bias		Misclassification bias			Reporting bias		Confounding bias	
	Exclusion of abnormal pre-radiotherapy TFT	Exclusion of prior thyroid disease	Exclusion of secondary hypothyroidism	Regular TFT monitoring after radiotherapy	Adequate follow-up duration (median >3 years)	Systematic evaluation of DVH parameter(s)	Systematic evaluation of cut-off value(s)	Multivariable adjustment for clinical factor(s)	Multivariable adjustment for thyroid volume
**Studies on NTCP model or radiation dosimetric nomograms (n=10)**									
Boomsma 2012									
Bakhshandeh 2012									
Ronjom 2013									
Ronjom 2015*									
Luo 2017									
Luo 2018									
Prpic 2019									
Nowicka 2020*									
Kamal 2020*									
Zhu 2021									

**External validation studies*
Abbreviations: DVH, dose-volume histogram; NTCP, normal tissue complication probability, TFT, thyroid function test.
