# Supplementary material for: Dose-volume predictors of post-radiation primary hypothyroidism in head and neck cancer: A systematic review
Source: Clin Transl Radiat Oncol. 2022 Jan 24;33:83–92. doi: 10.1016/j.ctro.2022.01.001 (PMC8807951; doi:10.1016/j.ctro.2022.01.001)
Supplement: Supplementary data 2 [file mmc2.pdf]

Supplementary Figure 2

Forest plot showing the association of thyroid volume with post-radiation hypothyroidism, stratified by median follow-up durations. Studies were included only if the reported odds ratios were adjusted for at least one radiation dose-volume parameter in multivariable analyses.

Study or Subgroup	log[Odds Ratio]	SE	Total	Total	Weight	Odds Ratio IV, Random, 95% CI	Year
**1.1.1 Median follow-up duration <3 years**							
Boomsma 2012	-0.19116051	0.05581884	105	0	9.7%	0.83 [0.74, 0.92]	2012
Ronjom 2013	-0.28768207	0.07389796	203	0	6.9%	0.75 [0.65, 0.87]	2013
Ronjom 2015	-0.28768207	0.1189475	198	0	3.3%	0.75 [0.59, 0.95]	2015
Nowicka 2020	-0.15082289	0.04162032	108	0	12.7%	0.86 [0.79, 0.93]	2020
Zhou 2020	-0.11653382	0.03174851	206	0	15.3%	0.89 [0.84, 0.95]	2020
**Subtotal (95% CI)**			**820**	**0**	**47.9%**	**0.84 [0.79, 0.89]**	
Heterogeneity: Tau² = 0.00; Chi² = 6.36, df = 4 (P = 0.17); I² = 37%							
Test for overall effect: Z = 5.71 (P < 0.00001)							
**1.1.2 Median follow-up duration >=3 years**							
Akgun 2014	-0.188	0.1001	100	0	4.4%	0.83 [0.68, 1.01]	2014
Chyan 2014	-0.05129329	0.04848051	107	0	11.2%	0.95 [0.86, 1.04]	2014
Peng 2020	-0.0618754	0.01628864	545	0	19.1%	0.94 [0.91, 0.97]	2020
Zhu 2021	-0.04709161	0.02352344	244	0	17.4%	0.95 [0.91, 1.00]	2021
**Subtotal (95% CI)**			**996**	**0**	**52.1%**	**0.94 [0.92, 0.97]**	
Heterogeneity: Tau² = 0.00; Chi² = 1.97, df = 3 (P = 0.58); I² = 0%							
Test for overall effect: Z = 4.59 (P < 0.00001)							
**Total (95% CI)**			**1816**	**0**	**100.0%**	**0.89 [0.85, 0.93]**	
Heterogeneity: Tau² = 0.00; Chi² = 23.68, df = 8 (P = 0.003); I² = 66%							
Test for overall effect: Z = 5.03 (P < 0.00001)							
Test for subgroup differences: Chi² = 11.96, df = 1 (P = 0.0005), I² = 91.6%							

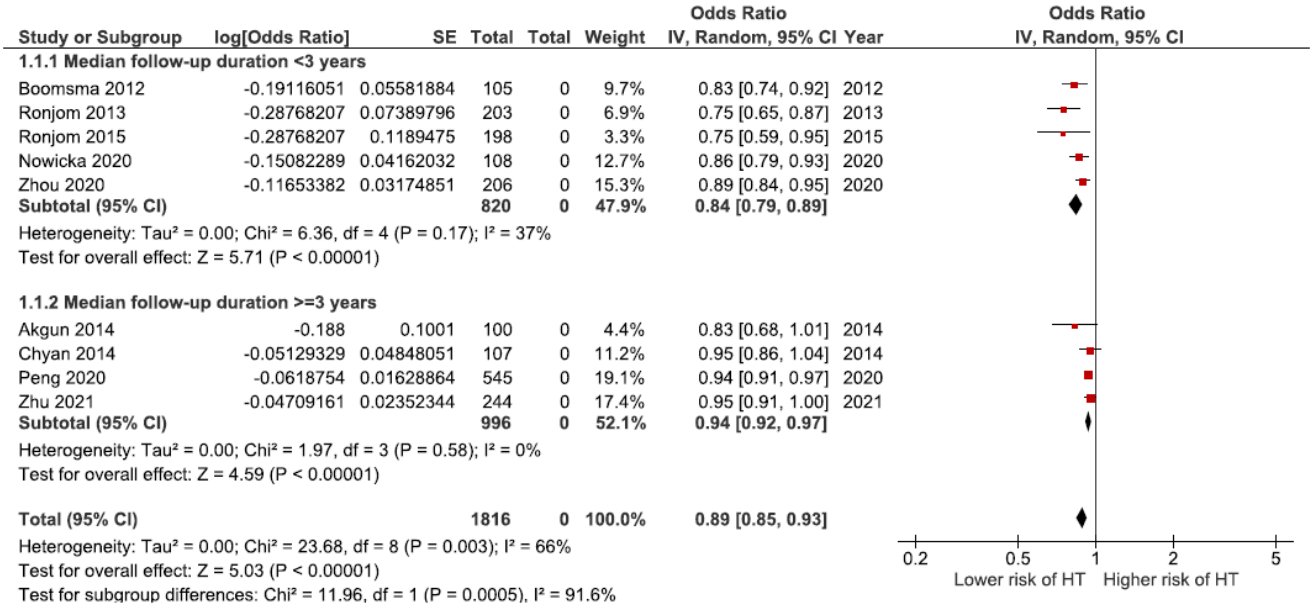

Abbreviations: CI, confidence interval; HT, hypothyroidism; IV, inverse variance; SE, standard error.
