# Supplementary material for: Dose-volume predictors of post-radiation primary hypothyroidism in head and neck cancer: A systematic review
Source: Clin Transl Radiat Oncol. 2022 Jan 24;33:83–92. doi: 10.1016/j.ctro.2022.01.001 (PMC8807951; doi:10.1016/j.ctro.2022.01.001)
Supplement: Supplementary data 3 [file mmc3.docx]

**Supplementary Table 1**

Search strategy for the current systematic review.

| **Search number** | **Query** | **Search Details** |
| --- | --- | --- |
| 16 | #13 and #14 and #15 | ("thyroid"[Title/Abstract] OR "hypothyroid*"[Title/Abstract]) AND ("radiation"[Title/Abstract] OR "radiotherapy"[Title/Abstract] OR "IMRT"[Title/Abstract]) AND ("head"[Title/Abstract] OR "neck"[Title/Abstract] OR "nasopharyn*"[Title/Abstract] OR "oropharyn*"[Title/Abstract] OR "hypopharyn*"[Title/Abstract] OR "laryn*"[Title/Abstract] OR "squamous"[Title/Abstract]) |
| 15 | #6 or #7 or #8 or #9 or #10 or #11 or #12 | "head"[Title/Abstract] OR "neck"[Title/Abstract] OR "nasopharyn*"[Title/Abstract] OR "oropharyn*"[Title/Abstract] OR "hypopharyn*"[Title/Abstract] OR "laryn*"[Title/Abstract] OR "squamous"[Title/Abstract] |
| 14 | #3 or #4 or #5 | "radiation"[Title/Abstract] OR "radiotherapy"[Title/Abstract] OR "IMRT"[Title/Abstract] |
| 13 | #1 or #2 | "thyroid"[Title/Abstract] OR "hypothyroid*"[Title/Abstract] |
| 12 | squamous[Title/Abstract] | "squamous"[Title/Abstract] |
| 11 | laryn*[Title/Abstract] | "laryn*"[Title/Abstract] |
| 10 | hypopharyn*[Title/Abstract] | "hypopharyn*"[Title/Abstract] |
| 9 | oropharyn*[Title/Abstract] | "oropharyn*"[Title/Abstract] |
| 8 | nasopharyn*[Title/Abstract] | "nasopharyn*"[Title/Abstract] |
| 7 | neck[Title/Abstract] | "neck"[Title/Abstract] |
| 6 | head[Title/Abstract] | "head"[Title/Abstract] |
| 5 | IMRT[Title/Abstract] | "IMRT"[Title/Abstract] |
| 4 | radiotherapy[Title/Abstract] | "radiotherapy"[Title/Abstract] |
| 3 | radiation[Title/Abstract] | "radiation"[Title/Abstract] |
| 2 | hypothyroid*[Title/Abstract] | "hypothyroid*"[Title/Abstract] |
| 1 | thyroid[Title/Abstract] | "thyroid"[Title/Abstract] |
