# Supplementary material for: Dose-volume predictors of post-radiation primary hypothyroidism in head and neck cancer: A systematic review
Source: Clin Transl Radiat Oncol. 2022 Jan 24;33:83–92. doi: 10.1016/j.ctro.2022.01.001 (PMC8807951; doi:10.1016/j.ctro.2022.01.001)
Supplement: Supplementary data 4 [file mmc4.docx]

**Supplementary Table 2**
Relationship of clinical factors with post-radiation hypothyroidism in patients with head and neck cancer. Studies were listed only if the reported relative effects were adjusted for at least one radiation dose-volume parameter in multivariable analyses.

| **Clinical factor** | **Studies finding increased risk of post-radiation hypothyroidism** | **Studies finding no association with post-radiation hypothyroidism** |
| --- | --- | --- |
| Age | Diaz 2010 (Young age)  Zhai 2017 (Young age)  Sommat 2017 (Young age)  Zhu 2021 (Young age) | Chyan 2014  Sachev 2017  Huang 2019  Boomsma 2012  Ronjom 2013  Ronjom 2015  Nowicka 2020 |
| Sex | Zhu 2021 (Female)  Luo 2017/2018 (Female) | Diaz 2010  Chyan 2014  Sachev 2017  Zhai 2017  Lertbutsayanukul 2018  Huang 2019  Ronjom 2013  Ronjom 2015  Nowicka 2020 |
| Smoking history | - | Nowicka 2020 |
| Thyrotoxicosis history | - | Zhai 2017 |
| Ideal body weight | - | Chyan 2014 |
| Tumor site | - | Ronjom 2013  Ronjom 2015 |
| Stage | Sommat 2017 (T3-4)  Zhou 2020 (N2-3) | Diaz 2010  Huang 2019  Ronjom 2013  Ronjom 2015 |
| Surgery | - | Ronjom 2013  Ronjom 2015  Nowicka 2020 |
| Chemotherapy | Luo 2017/2018 | Sachev 2017  Ronjom 2013  Ronjom 2015 |
| High pre-treatment TSH level | Lertbutsayanukul 2018  Ronjom 2015 | Ronjom 2013  Nowicka 2020 |

*Abbreviation: TSH, thyroid stimulating hormone.*
