# Supplementary material for: Dose-volume predictors of post-radiation primary hypothyroidism in head and neck cancer: A systematic review
Source: Clin Transl Radiat Oncol. 2022 Jan 24;33:83–92. doi: 10.1016/j.ctro.2022.01.001 (PMC8807951; doi:10.1016/j.ctro.2022.01.001)
Supplement: Supplementary data 5 [file mmc5.docx]

**Supplementary Table 3**

Four NTCP models for post-radiation hypothyroidism in head and neck cancer patients.

| Bakhshandeh 2012 | NTCP = $\left( \frac{1}{\sqrt{2}\pi} \right)\int_{-\infty}^{t} \exp({-t}^{2}/2) dt$, in which t= (D-D50)/(m*D50), where D is the thyroid mean dose, m=0.26 and D50 = 44.1 |
| --- | --- |
| Boomsma 2012 | NTCP = ${(1+e^{-S})}^{-1}$, in which S = 0.011 + (0.062*Dmean) + (-0.19 * thyroid volume) |
| Ronjom 2013 | NTCP = ${(1+e^{-S})}^{-1}$, in which S = -2.019 + (0.0821*Dmean) + (-0.189 * thyroid volume) |
| Luo 2018 | NTCP = ${(1+e^{-S})}^{-1}$, in which S = -2.695 + (0.05*V50) + (-0.026*Pmax) + (1.280 if female) + 2.902 if chemotherapy used) |

**Abbreviations: Dmean, mean thyroid dose; NTCP, normal tissue complication probability; Pmax, maximal pituitary dose.*
