# Supplementary material for: Dose-volume predictors of post-radiation primary hypothyroidism in head and neck cancer: A systematic review
Source: Clin Transl Radiat Oncol. 2022 Jan 24;33:83–92. doi: 10.1016/j.ctro.2022.01.001 (PMC8807951; doi:10.1016/j.ctro.2022.01.001)
Supplement: Supplementary data 6 [file mmc6.docx]

**Supplementary Table 4**

Three nomograms for post-radiation hypothyroidism in head and neck cancer patients.

| Luo 2016 | 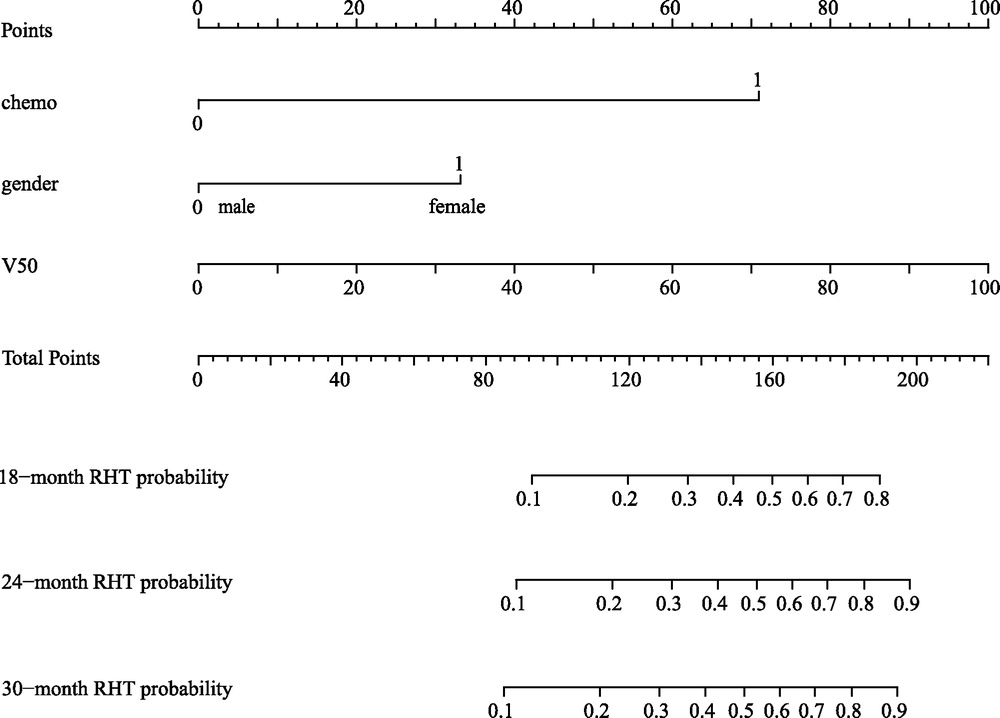 |
| --- | --- |
| Prpic 2019 | Hypothyroidism risk score = Log(10) Dmin x 2.286 – Log(10) TV x 1.165 |
| Zhu 2021 | **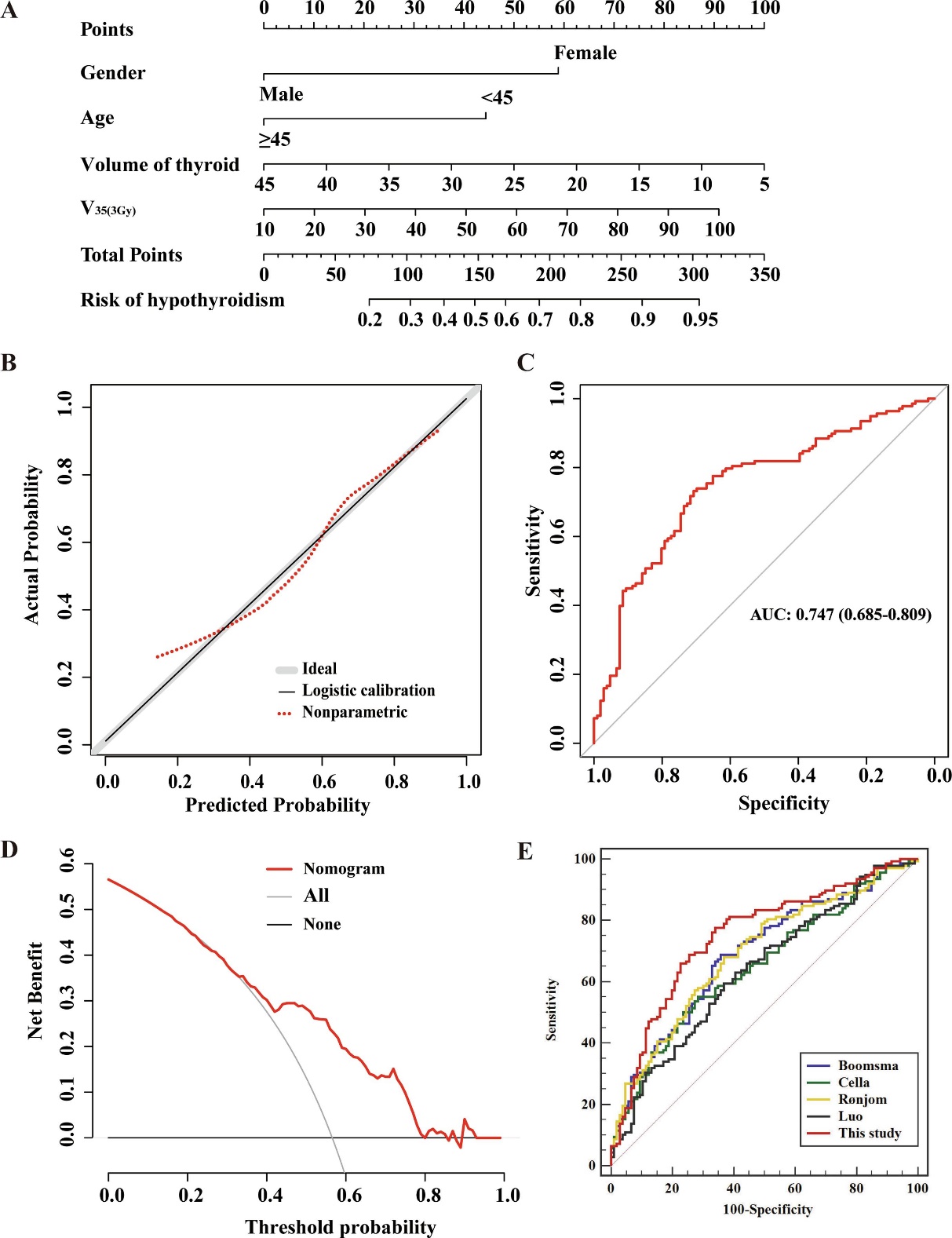** |

**Abbreviations: Chemo, chemotherapy; Dmin, minimal thyroid dose; RHT, radiation-associated hypothyroidism; TV, thyroid volume.*
